# Impact of Roux-en-Y Gastric Bypass and Vertical Gastrectomy on weight loss: a retrospective and longitudinal study in the State of Paraná, Brazil

**DOI:** 10.1590/0100-6991e-20233431-en

**Published:** 2023-01-24

**Authors:** FERNANDA PEREIRA GAMBA, BRUNA SCHUMAKER SIQUEIRA, RICARDO SHIGUEO TSUCHIYA, TOMAZ MASSAYUKI TANAKA, SABRINA GRASSIOLLI

**Affiliations:** 1 - Universidade Estadual do Oeste do Paraná, Programa de pós-graduação em biociências e saúde - Cascavel - PR - Brasil; 2 - Gastroclínica Cascavel, Cirurgia Bariátrica - Cascavel - PR - Brasil

**Keywords:** Obesity, Bariatric Surgery, Men, Women, Obesidade, Cirurgia Bariátrica, Homens, Mulheres

## Abstract

**Aim:**

to compare the impact of Roux’s Y Gastric Bypass (RYGB) and Sleeve Gastrectomy (SG) techniques on body weight reduction over 1 and 5 years after bariatric surgery in obese patients in the state of Paraná.

**Methods:**

longitudinal and retrospective study, conducted between January 2010 and December 2013, with 737 patients of both sexes submitted to RYGB or SG and evaluated in the preoperative, 1 and 5 years after bariatric surgery (BS). Age, height, body weight, Body Mass Index (BMI), biochemical and pressure parameters were recorded.

**Results:**

of the total of patients, men represented lower frequency, were slightly older, with higher body weight, BMI and worse metabolic and pressure conditions than women in pre-BS (p<0.05). Regardless of sex, RYGB and SG were effective in promoting body weight reduction and BMI in 1 and 5 years after BS; the RYGB technique had greater impact on these variables in both sexes (p<0.05). The highest percentage of lost weight was observed in women who underwent the RYGB technique in the first year after BS. Five years after BS, the RYGB technique promoted a higher rate of body weight reduction in men and women compared to the SG technique (p<0.05).

**Conclusion:**

regardless of sex, the RYGB technique promotes a higher degree of body weight reduction and BMI over time compared to the SG; having its biggest impacts in the 1 year after BS, especially in women.

## RESUMO

**Objetivo:**

comparar o impacto das técnicas Bypass Gástrico em Y de Roux (BGYR) e Gastrectomia Vertical (GV) na redução do peso corporal ao longo de 1 e 5 anos após a cirurgia bariátrica em pacientes obesos no Estado do Paraná.

**Métodos:**

estudo longitudinal e retrospectivo, realizado entre janeiro de 2010 a dezembro de 2013, com 737 pacientes de ambos os sexos submetidos a BGYR ou GV e avaliados no pré-cirúrgico, 1 e 5 anos após Cirurgia Bariátrica (CB). Foram registrados idade, estatura, peso corporal, Índice de Massa Corpórea (IMC), parâmetros bioquímicos e pressóricos.

**Resultados:**

do total de pacientes, homens representavam menor frequência, eram ligeiramente mais velhos, com maior peso corporal, IMC e pior condição metabólica e pressórica que mulheres no pré-CB (p<0,05). Independente do sexo, BGYR e GV foram efetivas em promover redução do peso corporal e IMC em 1 e 5 anos pós-CB; tendo a técnica de BGYR maior impacto nestas variáveis em ambos os sexos (p<0,05). A maior porcentagem de peso perdido foi observada em mulheres submetidas a técnica da BGYR no primeiro ano após a CB. Cinco anos após a CB, a técnica de BGYR promoveu maior taxa de redução de peso corporal em homens e mulheres comparado a técnica da GV (p<0,05).

**Conclusão:**

independente do sexo a técnica de BGYR promove maior grau de redução do peso corporal e IMC ao longo do tempo em comparação com a GV; tendo seus maiores impactos no 1 ano pós-CB sobretudo em mulheres.

## INTRODUCTION

Overweight and obesity affect more than 30% of the world’s adult population. In 2025, estimates indicate that severe obesity, that is, individuals with a Body Mass Index (BMI) ≥40Kg/m^2^, may affect 6% of men and 8% of women worldwide^1^. In Brazil, data recorded by the Food and Nutrition Surveillance System (SISVAN) of the Ministry of Health showed that in 2019, more than 65% of the Brazilian adult population was above adequate body weight. Of these, more than 3% have grade III obesity, that is, BMI ≥40Kg/m^2^, a prerequisite for bariatric surgery (BS). In the State of Paraná, almost 70% of the adult population is overweight and more than 4% of these individuals are considered obese grade III^2^.

The excessive accumulation of white adipose tissue (WAT), typical of obese individuals, is linked to a chronic pro-inflammatory condition, which reduces the action of insulin and causes glucose intolerance, dyslipidemia, and arterial hypertension, factors comprising the so-called Metabolic Syndrome (MS). Obesity and MS are central elements for the installation of chronic noncommunicable diseases (NCDs), such as diabetes and cardiovascular diseases (CVD)^3^. In Brazil, 72% of the causes of death and 75% of health care expenditures in the Unified Health System (SUS) are related to (NCDs), especially diabetes and CVD, a situation that is reflected in Paraná^2^ ^,^ ^4^.

Therefore, the search for strategies that enable the reduction of WAT and promote the restoration of glycemic and lipid homeostasis and cardiovascular health are fundamental to guarantee the quality of life of these patients and to avoid the onset of NCDs^5^. In this context, BS is capable of promoting, in addition to significant, rapid, and sustainable weight loss, improvement in glycemic control, dyslipidemia, normalizing blood pressure, and reducing the risks of CVD and diabetes^6^ ^,^ ^7^.

Every year, around 635,000 BSs are performed worldwide. Brazil ranks second in the number of BSs performed and has the largest number of bariatric surgeons in the world, followed by the United States. Between 2003 and 2018, the number of BSs performed in Brazil increased more than sixfold^8^ ^-^ ^10^. According to Marchesini, in 2021 Brazil had 4.9 million people eligible for BS, of whom more than 210 thousand are in the State of Paraná. It is important to emphasize that in Brazil, more than 90% of BSs are performed by private health services^11^ ^-^ ^13^.

Roux-en-Y Gastric Bypass (RYGB) and Sleeve Gastrectomy (SG) are the two most commonly performed BS techniques worldwide^14^. RYGB corresponds to 75% of BSs performed in Brazil and is considered the gold standard for weight loss and restoration of glycemic homeostasis. SG is considered a simpler technique, with fewer late complications, such as hernias and ulcerations, and less impact on the absorption of nutrients, such as iron, calcium and vitamins, which may explain the preference for this technique in some countries, such as the United States, Scotland, and Germany^9^ ^,^ ^13^ ^-^ ^15^.

As pointed out by 2018 data from the Federation for the Surgery of Obesity and Metabolic Disorders (IFSO), Brazil still does not have a consolidated national system for registering BS, and among the 51 countries that participated in the study, it is one of those that did not provide complete information on the profile of patients undergoing BS. Only one hospital in the capital of Paraná participated in the IFSO 2018 records^8^. Additionally, there are few Brazilian studies comparing the long-term impact of SG and RYGB techniques, mainly with regard to BMI reduction, weight loss, and gender influence^10^ ^,^ ^16^. Therefore, the expansion of national publications on the effects of BS, especially in the long term, has significant relevance for the follow-up and management of these patients.

Thus, in the present study, we compared the impact of RYGB and SG techniques on body weight loss and BMI reduction over one and five years after BS, considering the influence of sex.

## METHODS

The present study is a longitudinal and retrospective one, in which data were collected from the medical records of 737 patients of both sexes, who underwent RYGB or SG in a private hospital in Western Paraná from January 2010 to December 2013.

The Ethics in Human Research Committee of the State University of Western Paraná (UNIOESTE-CAAE) approved this study under protocol number 80388317.2.0000.0107, following the criteria previously established by Ordinance 425 of the Brazilian Ministry of Health^17^.

The sampled patients were between 18 and 65 years old, with BMI ≥40Kg/m^2^, or BMI >35Kg/m^2^ associated with two or more comorbidities and clinical treatment failures conducted for more than two years. Both surgical procedures, RYGB and SG, were performed by the same surgical team and with the same techniques.

Of the 737 patients, we recorded sex (male or female), age (years), height (m), body weight (Kg), and BMI (Kg/m^2^), obtained in three moments: before BS, one year, and five years after BS. When present, in the pre-BS we also recorded Systolic (SBP; mmHg) and diastolic (DBP; mmHg) blood pressure values, as well as plasmatic biochemical values (mg/dL) of glucose, triglycerides, total cholesterol, HDL, and LDL. These data were categorized as Adequate or Altered according to the recommendations of the Federal Council of Medicine. The respective numbers for these data are indicated in the tables.

Body weight and BMI values were evaluated pre-BS and one and five years after BS, comparing the effects of the RYGB and SG techniques, as well as the influence of sex. The data obtained were tabulated in Microsoft Excel^®^ spreadsheets and submitted to statistical assumptions of normality (Shapiro-Wilk Test) and homoscedasticity (Cochran Test), followed by association analysis using the T-Test for unpaired samples.

When the assumptions of normality and homoscedasticity were not accepted, the Mann-Whitney U Test was applied. To assess the existence of an association between the different variables, the PERMANOVA Test was applied. Statistical analyzes were performed using XLStat Version 19.4 (ADDINSOFT, 2018) and the R computational program (R Development Core Team, 2019). We adopted p<0.05 as a significance criterion in all analyses.

## RESULTS

Of the patients undergoing BS, 589 (80%) underwent RYGB and 148 (20%) SG. The profile of obese patients before BS is shown in Table 1. The mean age of women was 39.3 years, slightly lower than men (p<0.0001). Furthermore, as a consequence of sexual dimorphism, women had lower body weight and height compared with men (p<0.0001). However, men had higher BMI values than women (p<0.0001).

**Table 1:** Anthropometric data, blood pressure, and plasma biochemical parameters in pre-BS women and men.

	Women	Men	p-value
Age (years old)	39.3 ± 11.9 (n=533)	40.9 ± 11.9 (n=204)	<0.0001
Body Weight (Kg)	106.4 ± 16.7 (n=533)	129.1 ± 21.7 (n=204)	<0.0001
Height (cm)	162.6 ± 6.7 (n=533)	176.5 ± 6.9 (n=204)	<0.0001
BMI (Kg/m^2^)	40.1 ± 5.1 (n=533)	41.7 ± 6.2 (n=204)	<0.0001
Glycemia (mg/dL)	96 [88.8 - 111.4] (n=111)	103 [92.2 - 122.0] (n=48)	0.108^#^
Triglycerides (mg/dL)	155.4 ± 81.4 (n=81)	200.3 ± 84.5 (n=38)	0.007
Total Cholesterol (mg/dL)	198.2 ± 40.8 (n=82)	183.7 ± 34.5 (n=39)	0.056
LDL (mg/dL)	118.0 ± 35.8 (n=80)	106.9 ± 25.2 (n=38)	0.087
HDL (mg/dL)	46 [39.5 - 54.1] (n=80)	38.5 [33.5 - 44.9] (n=38)	<0.0001^#^
SBP (mmHg)	130 [120.0 -140.0] (n=467)	140 [130.0 -150.0] (n=174)	<0.0001^#^
DBP (mmHg)	80 [70.0 - 90.0] (n=406)	87 [80.0 - 92.0] (n=173)	<0.0001^#^

Data expressed as mean ± SD; Student’s t-test p-value or mean and interquartile range [1Q and 3Q]; #p-value Mann-Whitney U.

We observed no significant differences in blood glucose and LDL concentrations between sexes (p>0.05). However, plasma triglyceride levels were significantly higher in men (p=0.007).

In contrast, total and HDL cholesterol values were elevated in women compared with men. Males had higher DBP and SBP values compared with females (Table 1; p<0.0001).

Table 2 brings the clinical characteristics of the patients before BS in both sexes categorized as adequate and altered. In men, there is a higher frequency of altered values of SBP and DBP (p<0.001), glucose (p=0.030), and triglycerides (p=0.022) in relation to women. On the other hand, women have more frequent elevation of HDL than men (p=0.002). The frequency of total and LDL cholesterol values were similar between sexes (p>0.05).

**Table 2:** Categorization of clinical and biochemical variables in pre-BS women and men.

		Women		Men		
Variable	Category	AF	RF (%)	AF	RF (%)	p-value*
SBP	Altered	179	38.33	103	59.20	
	Adequate	288	61.67	71	40.80	<0.0001
DBP	Altered	164	35.19	93	53.76	
	Adequate	302	64.81	80	46.24	<0.0001
Glycemia	Altered	44	39.64	28	58.33	
	Adequate	67	60.36	20	41.67	0.030
Triglycerides	Altered	33	40.74	24	63.16	
	Adequate	48	59.26	14	36.84	0.022
Total cholesterol	Altered	77	93.90	36	92.31	
	Adequate	5	6.10	3	7.69	0.741
HDL	Altered	55	68.75	15	39.47	
	Adequate	25	31.25	23	60.53	0.002
LDL	Altered	55	68.75	25	65.79	
	Adequate	25	31.25	13	34.21	0.748

*Chi-square test of independence; AF: absolute frequency; RF: relative frequency.

**Figure 1:**
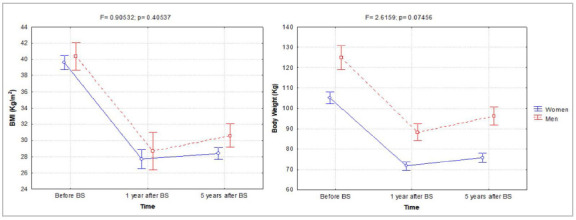
Changes in BMI and body weight induced by BS in women and men over time. Data are mean and confidence interval (95% CI).

The influence of BS on body weight and BMI in men and women over time is shown in Figure 1. BMI was significantly influenced by the time factor (F=255.7; p=0.000), but not by the sex factor (F=0.9; p=0.405). Thus, for both sexes, we observed a significant reduction in BMI at one and five years after BS, with a more pronounced drop in the first year after BS (Figure 1A). In contrast, body weight was influenced by sex (F=55.3; p=0.000) and time (F=803.7; p=0.000), but without interaction (F=2.6; p=0.075). Thus, women have lower body weight over time compared with men. In both sexes, we found a marked loss of body weight one year after BS, which was relatively preserved five years after BS (Figure 1B).

**Figure 2:**
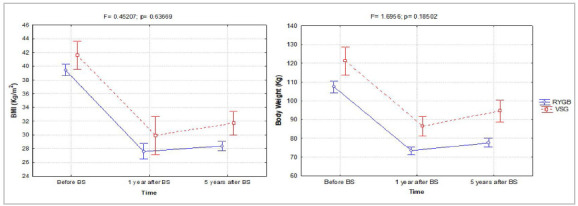
Comparative effects of changes in BMI and body weight induced by RYGB and SG. Data are means and confidence intervals (95% CI).

Figure 2 shows the comparative effect between the RYGB and SG techniques on BMI and body weight. The type of surgical technique significantly influences the magnitude of the decrease in body weight (F=20.8, p=0.000) and the reduction in BMI (F=7.6, p=0.007). The time factor also significant influences body weight (F=563.7; p=0.000) and BMI (F=188.5; p=0.000), without interaction between the factors. In this sense, RYGB promoted a greater degree of reduction in BMI (Figure 2A) and body weight (Figure 2B) compared with SG. One year after RYGB and SG, patients showed a marked reduction in BMI and body weight compared with the period before BS, the effect being relatively preserved after five years.

**Figure 3:**
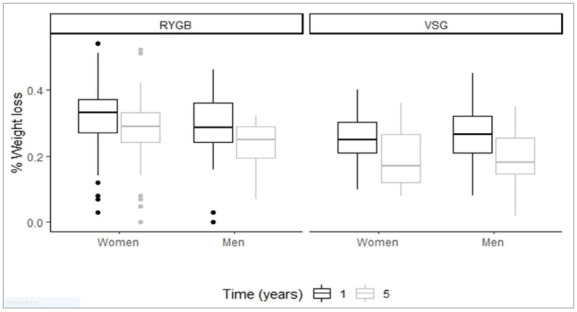
Percentage of weight lost at one and 5 years after different types of BS in men and women. Data are mean and confidence interval (95% CI).

Finally, we compared the percentage of weight lost at one and five years after BS, considering type of technique and sex (Figure 3). We found that one year after BS, women undergoing RYGB showed a higher percentage of weight loss compared with women and men undergoing SG. In addition, five years after BS, women and men undergoing RYGB maintained a higher percentage of weight loss compared with those undergoing SG.

## DISCUSSION

Paraná is the state in which most BSs are performed in Brazil, the majority in private hospitals and whose impacts on health, especially in the long term, are little known. Therefore, our study presents unpublished data regarding the impact of RYGB and SG techniques on weight loss and BMI of obese patients from Paraná, over one and five years.

RYGB and SG procedures represent almost 85% of all BSs in the world, with frequencies varying according to different countries^8^ ^,^ ^9^ ^,^ ^15^. In our sample, more than 80% of patients underwent RYGB, the majority of patients being female, confirming data previously published in other regions of Brazil and the world9,18-20. In contrast to Brazil, ISFO world records (2018) indicated that in the last two years there have been increases in SG in relation to RYGB in many countries^8^.

The age profile and BMI values observed in our patients in the pre-BS period were also similar to other national and international studies^21^ ^,^ ^22^. Considering pre-BS data, men had higher BMI values and a greater presence of glycemic, lipid, and blood pressure alterations compared with women. Obesity reduces male testosterone levels, a factor that is related to a higher incidence of atherosclerosis, hypertension, hyperinsulinemia, dyslipidemia, and diabetes^23^ ^-^ ^25^. These findings indicate the need for greater attention to the health condition of the male population of Paraná, especially the obese.

The benefits of BS for health are widely recognized, especially in morbidly obese individuals, in whom there is a significant reduction in body weight and adiposity and improvement in glycemic, lipid, and blood pressure control^26^. Our findings confirm these, as regardless of gender, in both techniques (RYGB and SG) there was a significant reduction in body weight and BMI over time, especially in the first year after BS. We could not evaluate metabolic and blood pressure aspects over time, confirming the need to expand long-term records, an aspect also highlighted by the IFSO 2018 records^8^. A recent Brazilian study carried out by Schiavon et al. (2020) showed that RYGB was effective in controlling blood pressure in men and women three years after BS^27^. Similarly, a recent retrospective study with more than 5,000 patients undergoing BS showed that RYGB and SG are effective in promoting diabetes remission, the former displaying better results^28^.

Regardless of sex, our data showed that both the reduction in body weight and the decrease in BMI were more pronounced in patients who underwent the RYGB technique, at one and also at five years after the procedure. In contrast, Palacio et al. showed in 2019 that six months after BS, the percentage of fat mass loss was similar in patients undergoing RYGB and SG. Other studies found no differences in terms of weight reduction between RYGB and SG one year after BS^29^ ^,^ ^30^. On the other hand, a metanalysis performed by Li et al. (2016) showed a higher percentage of weight lost in patients undergoing RYGB compared with patients undergoing SG^31^, corroborating our findings.

Hayoz et al., in 2018, emphasized that studies on BS with five years of follow-up are scarce, making it difficult to analyze the durability of these effects^32^. Therefore, the results of the relative maintenance of the effects of weight loss and BMI five years after BS are unprecedented for this population and reveal a longer-lasting effect promoted by the RYGB technique.

Sex is a determining factor in metabolism and responses to weight reduction procedures^33^. In the first year after BS, we found the highest rate of weight loss in women undergoing RYGB, confirming the study by Olbers et al. (2006), who showed a greater reduction in visceral WAT in women after RYGB compared with women who underwent SG^34^. Korner et al. (2008) found no difference in the reduction of visceral WAT in men with RYGB and the control group^35^.

Five years after BS, our findings reveal that, regardless of sex, RYGB promotes greater weight loss compared with SG, confirming the greater efficacy of this technique for long-term weight loss^35^. RYGB induces anti-inflammatory responses and stimulates the release and action of incretins (intestinal hormones), particularly glucagon-like peptide 1 (GLP1)^36^ ^,^ ^37^. A recent study in mice indicated that RYGB is more efficient than SG in preventing weight gain induced by a high-fat diet after BS, indicating a protective effect against excess lipids in the diet^37^. On the other hand, a Brazilian study carried out with bariatric patients in the northeast region indicated that RYGB causes a more pronounced decrease in minerals such as iron and zinc compared with SG two years after BS^38^. Additionally, many bariatric patients regain weight over time^39^, indicating the need for more careful monitoring of bariatric patients.

Taken together, the results of the present study show that in a sample of obese individuals from Paraná, men have a higher BMI and a worse metabolic state than women at the pre-surgical moment, and that regardless of sex, RYGB is more effective in promoting body weight reduction and BMI over time, with the strongest effect in women in the first post-BS year.

## References

[1] NCD Risk Factor Collaboration (NCD-RisC) (2016). Trends in adult body-mass index in 200 countries from 1975 to 2014: a pooled analysis of 1698 population-based measurement studies with 19·2 million participants. Lancet.

[2] SISVAN - Sistemas Informatizados https://sisaps.saude.gov.br/sisvan/relatoriopublico/index.

[3] De Lorenzo A, Soldati L, Sarlo F, Calvani M, Di Lorenzo N, Di Renzo L (2016). New obesity classification criteria as a tool for bariatric surgery indication. World J Gastroenterol.

[4] Paraná (2018). Secretaria de Estado da Saúde do Paraná. Superintendência de Atenção à Saúde.P223l Linha guia de hipertensão arterial / SAS. -.

[5] Lavie CJ, De Schutter A, Parto P, Jahangir E, Kokkinos P, Ortega FB (2016). Obesity and Prevalence of Cardiovascular Diseases and Prognosis-The Obesity Paradox Updated. Prog Cardiovasc Dis.

[6] Sjöström L, Lindroos AK, Peltonen M, Torgerson J, Bouchard C, Carlsson B (2004). Lifestyle, diabetes, and cardiovascular risk factors 10 years after bariatric surgery. N Engl J Med.

[7] Buchwald H, Avidor Y, Braunwald E, Jensen MD, Pories W, Fahrbach K (2004). Bariatric surgery a systematic review and meta-analysis. JAMA.

[8] Welbourn R, Hollyman M, Kinsman R, Dixon J, Liem R, Ottosson J (2019). Bariatric Surgery Worldwide Baseline Demographic Description and One-Year Outcomes from the Fourth IFSO Global Registry Report 2018. Obes Surg.

[9] Angrisani L, Santonicola A, Iovino P, Vitiello A, Zundel N, Buchwald H (2017). Bariatric Surgery and Endoluminal Procedures IFSO Worldwide Survey 2014. Obes Surg.

[10] Sociedade Brasileira de Cirurgia Bariátrica e Metabólica Home. Cirurgia Bariátrica. Notícias. Notícias destaque. Cirurgia bariátrica cresce 84,73% entre 2011 e 2018. Cirurgias bariátricas realizadas em 2018 representam 0,47% da população elegível ao procedimento. Custo da obesidade.

[11] Cazzo E, Ramos AC, Pareja JC, Chaim EA (2018). Nationwide Macroeconomic Variables and the Growth Rate of Bariatric Surgeries in Brazil. Obes Surg.

[12] Agência Nacional de Saúde Suplementar, Brasil Dados gerais: Beneficiários de planos privados de saúde, por cobertura assistencial (Brasil 2007-2017).

[13] Rasera I, Luque A, Junqueira SM, Brasil NC, Andrade PC (2017). Effectiveness and Safety of Bariatric Surgery in the Public Healthcare System in Brazil Real-World Evidence from a High-Volume Obesity Surgery Center. Obes Surg.

[14] Angrisani L, Santonicola A, Iovino P, Vitiello A, Higa K, Himpens J (2016). IFSO Worldwide. Survey.

[15] Angrisani L, Santonicola A, Iovino P, Formisano G, Buchwald H, Scopinaro N (2015). Bariatric Surgery Worldwide 2013. Obes Surg.

[16] Pires Souto K, Meinhardt NG, de Azevedo Dossin I, Ramos MJ, Carnellos G, Mazzaferro C (2018). Revisional Malabsorptive Bariatric Surgery 29-Year Follow-up in a Brazilian Public Hospital. Obes Surg.

[17] Ministério da Saúde (2013). Portaria no 425, de 19 de março de 2013, estabelece regulamento técnico, normas e critérios para o Serviço de Assistência de Alta Complexidade ao Indivíduo com Obesidade.

[18] Fisher DP, Johnson E, Haneuse S, Arterburn D, Coleman KJ, O'Connor PJ (2018). Association between bariatric surgery and macrovascular disease outcomes in patients with type 2 diabetes and severe obesity. JAMA.

[19] Ferrannini E, Sironi AM, Iozzo P, Gastaldelli A. (2008). Intraabdominal adiposity, abdominal obesity, and cardiometabolic risk. European Heart Journal Supplements.

[20] Lyon M, Bashian C, Sheck C, Kushnir L, Slotman GJ (2019). Outcomes following laparoscopic Roux-en-Y gastric bypass (LRYGB) vary by sex Analysis of 83,059 women and men with morbid obesity. Am J Surg.

[21] de Melo MAF, dos Santos T, Godoy L, Silva K, Mezzomo TR, Zaparolli MR (2019). Efeito da redução de peso em pacientes submetidos à técnica do Bypass Gástrico em Y-de-Roux. Revista de Ciências Médicas.

[22] Sierzantowicz R, Lewko J, Hady HR, Kirpsza B, Trochimowicz L, Dadan J (2017). Effect of BMI on quality of life and depression levels after bariatric surgery. Adv Clin Exp Med.

[23] Lima N, Cavaliere H, Halpern A, Medeiros-Neto G (2000). A função gonadal do homem obeso. Arq Bras Endocrinol Metab.

[24] Escobar-Morreale HF, Santacruz E, Luque-Ramírez M, Botella Carretero JI (2017). Prevalence of 'obesity-associated gonadal dysfunction' in severely obese men and women and its resolution after bariatric surgery a systematic review and meta-analysis. Hum Reprod Update.

[25] Kolovou G, Bilianou H, Marvaki A, Mikhailidis DP (2011). Aging Men and Lipids. American Journal of Men's Health.

[26] Adams TD, Davidson LE, Litwin SE, Kim J, Kolotkin RL, Nanjee MN (2017). Weight and Metabolic Outcomes 12 Years after Gastric Bypass. N Engl J Med.

[27] Schiavon CA, Bhatt DL, Ikeoka D, Santucci EV, Santos RN, Damiani LP (2020). Three-Year Outcomes of Bariatric Surgery in Patients With Obesity and Hypertension A Randomized Clinical Trial. Ann Intern Med.

[28] Nudotor RD, Prokopowicz G, Abbey EJ, Gonzalez A, Canner JK, Steele KE (2021). Comparative Effectiveness of Roux-en Y Gastric Bypass Versus Vertical Sleeve Gastrectomy for Sustained Remission of Type 2 Diabetes Mellitus. J Surg Res.

[29] Palacio A, Quintiliano D, Lira I, Navarro P, Orellana V, Reyes A (2019). Cambios de la composición corporal en pacientes sometidos a cirugía bariátrica bypass gástrico y gastrectomía en manga [Changes in body composition in patients following bariatric surgery: gastric bypass and sleeve gastrectomy]. Nutr. Hosp.

[30] Yaghoubian A, Tolan A, Stabile BE, Kaji AH, Belzberg G, Mun E (2012). Laparoscopic Roux-en-Y gastric bypass and sleeve gastrectomy achieve comparable weight loss at 1 year. Am Surg.

[31] Li J, Lai D, Wu D (2016). Laparoscopic Roux-en-Y Gastric Bypass Versus Laparoscopic Sleeve Gastrectomy to Treat Morbid Obesity-Related Comorbidities a Systematic Review and Meta-analysis. Obes Surg.

[32] Hayoz C, Hermann T, Raptis DA, Brönnimann A, Peterli R, Zuber M (2018). Comparison of metabolic outcomes in patients undergoing laparoscopic roux-en-Y gastric bypass versus sleeve gastrectomy - a systematic review and meta-analysis of randomised controlled trials. Swiss Med Wkly.

[33] Escobar-Morreale HF, Santacruz E, Luque-Ramírez M, Botella Carretero JI (2017). Prevalence of 'obesity-associated gonadal dysfunction' in severely obese men and women and its resolution after bariatric surgery a systematic review and meta-analysis. Hum Reprod Update.

[34] Olbers T, Björkman S, Lindroos A, Maleckas A, Lönn L, Sjöström L (2006). Body composition, dietary intake, and energy expenditure after laparoscopic Roux-en-Y gastric bypass and laparoscopic vertical banded gastroplasty a randomized clinical trial. Ann Surg.

[35] Korner J, Punyanitya M, Taveras C, McMahon DJ, Kim HJ, Inabnet W (2008). Sex differences in visceral adipose tissue post-bariatric surgery compared to matched non-surgical controls. Int J Body Compos Res.

[36] Migliore R, Gentile JKA, Franca FT, Kappaz GT, Bueno-deSouza PMS, Assef JC (2018). Impact of bariatric surgery on the inflammatory state based on cpr value. ABCD, arq. bras. cir. dig.

[37] Stevenson M, Srivastava A, Lee J, Hall C, Palaia T, Lau R (2021). RYGB is more effective than VSG at protecting mice from prolonged high-fat diet exposure an occasion to roll up our sleeves?. Obes Surg.

[38] Ferraz ÁAB, Carvalho MRC, Siqueira LT, Santa-Cruz F, Campos JM (2018). Micronutrient deficiencies following bariatric surgery a comparative analysis between sleeve gastrectomy and Roux-en-Y gastric bypass. Rev Col Bras Cir.

[39] Goretti G, Marinari GM, Vanni E, Ferrari C (2020). Value-Based Healthcare and Enhanced Recovery After Surgery Implementation in a High-Volume Bariatric Center in Italy. Obes Surg.

